# Revisiting the dynamics of proteins during milk powder hydration using asymmetric flow field-flow fractionation (AF4)

**DOI:** 10.1016/j.crfs.2021.02.004

**Published:** 2021-02-23

**Authors:** Anouk Lie-Piang, Mats Leeman, Alejandra Castro, Erik Börjesson, Lars Nilsson

**Affiliations:** a Tetra Pak Processing Equipment, Ruben Rausings Gata, SE-221 86, Lund, Sweden; b SOLVE Research and Consultancy AB, Medicon Village, SE-223 81, Lund, Sweden; c Department of Food Technology, Engineering and Nutrition, Lund University, Getingevägen 60, 221 00, Lund, Sweden

**Keywords:** Milk powder, Reconstitution, Casein, Beta-casein, Asymmetrical flow field-flow fractionation, AF4

## Abstract

The dynamics of β-casein and casein micelles in the reconstitution of skim milk were revisited in this study. β-casein migrates into casein micelles upon an increase in temperatures due to an increase in the hydrophobic effect and lower calcium-phosphate cluster solubility. This process can be reversed upon cooling. These phenomena are well known in fresh milk and are not yet clearly established for reconstituted milk powder. As milk powder is commonly used as a functional ingredient in food products, it is of interest to investigate the migration of casein micelle β-casein to and from the serum phase in reconstituted milk. This study aimed to use asymmetrical flow field flow fractionation (AF4) in combination with various detectors to revisit the dynamics of β-casein when reconstituting skim milk at different temperatures. Fluorescence-labelled β-casein was added to fresh and reconstituted skim milk and rapid transport of β-casein into the outer shell of the casein micelles could be observed already after 5 ​min of reconstitution at 50 ​°C. This process stabilized after approximately 5 ​h, which indicates that an equilibrium of β-casein between the serum and the micellar phase was reached. Similar results were found for fresh milk. The apparent density of the casein micelles in the skim milk samples was also found to increase during reconstitution at 50 ​°C. During cold reconstitution of milk powders, the migration of β-casein to the serum was not observed. The results suggest that β-casein was already present in the serum phase upon reconstitution at 6 ​°C. When a sample was reconstituted for 180 ​min at 50 ​°C, the migration of β-casein back into the serum was observed upon cooling the same sample to 6 ​°C. The size of casein micelles in reconstituted milk at 6 ​°C was larger compared to reconstitution at 50 ​°C. With AF4 and the multi-detector approach, the change in concentration and size of casein micelles can be readily investigated and the migration of β-casein can be tracked simultaneously. Therefore, the method is a valuable tool for studies of the properties and changes in various milk samples.

## Highlights

• AF4 can be used to study the protein dynamics during skim milk reconstitution.
• Fluorescent labelling is used to track β-casein migration during reconstitution.
• β-casein migrates into the outer shell of casein micelles upon hot reconstitution.
• Migration of β-casein into the casein micelles increases their apparent density.
• Cold reconstitution yields larger casein micelles compared to hot reconstitution.

## Graphical abstract

**Figure:**
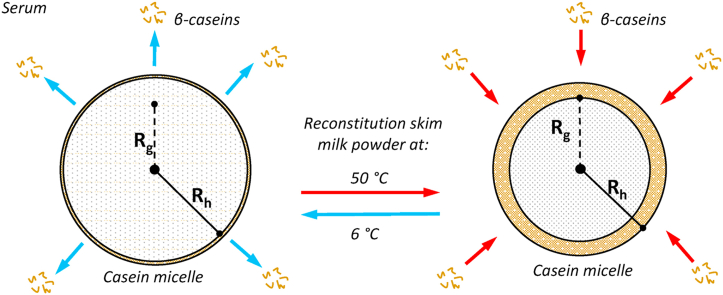

## 1 Introduction

The techno-functional properties of casein are often used in the food industry to provide thickening, gel formation, emulsification, and foaming. The casein can originate from their native environment, milk, in for example cheese production, but also as milk powders or as casein isolate powders. Dried milk or variants of it, enable the storage and transportation of perishable milk while maintaining the technical functionality of the product. The relation between modifications (e.g. heating, high pressure, additions of chelators, etc.) and the structure of milk has been studied before (Dalgleish and Corredig, 2012). Upon use, milk powder is reconstituted for specific food applications and the conditions under which the reconstitution takes place affect the properties of the final product. For example, the distribution of the milk protein β-casein between the casein micelles and serum phase is affected by the storage temperature and therefore results in stronger or weaker gels in cheeses (Ali et al., 1980). In addition, the acidification during cheese production alters the equilibrium of calcium between the micellar and serum phase, which influences the fluidity of melted cheese (Koutina et al., 2014). Therefore, understanding and tracking the properties of casein micelles in reconstituted skim milk is important for the understanding of its functionality in formulated food products.

Casein micelles in milk have proven to be a complex colloidal system. The casein micelle consists of four different types of casein proteins that are kept together by calcium-phosphate clusters in a structure that contains water channels (Dalgleish, 2011). The water channels allow loosely associated β-casein to leave or enter the micellar structure and, hence, the casein micelle is a highly dynamic structure. It has been shown in fresh milk, kept at 4 ​°C, that up to 60% of the original β-casein leaves the casein micelles and enters the serum phase. This could be reversed by returning the temperature of the milk to 37 ​°C (Creamer et al., 1977). A more recent study has shown that the amount of β-casein in the serum phase at 40 ​°C is approx. 25% of the corresponding amount at 10 ​°C (Liu et al., 2013). Two phenomena have been proposed as explanations for the observed behaviour. The solubility of calcium-phosphate clusters within casein micelles increases upon a decrease in temperature. This results in a breakage of the calcium phosphate bridges between β-casein and the micellar structure and, hence, β-casein is then retained as a result of the hydrophobic effect (Creamer et al., 1977; Downey and Murphy, 1970; Pierre and Brule, 1981). Since the hydrophobic effect decreases slightly at lower temperatures (Gill et al., 1976), the β-casein dissociates from the micelles. This is also manifested by β-casein in solution being present predominantly as a monomer at lower temperatures (de Kruif and Grinberg, 2002). The opposite applies for the solubility of calcium-phosphate clusters (Oldfield et al., 2005) and the hydrophobic effect at elevated temperatures (O’Connell et al., 2003). Nevertheless, most studies on the temperature-dependent dissociation/association behaviour of β-casein in relation to casein micelles were performed with fresh milk.

Casein micelle dynamics have also been studied in the powder manufacturing and reconstitution of skim milk powder (Martin et al., 2007). Using a combination of turbidity, SDS-page, and pH measurements, the authors observed an increase in the migration of β-casein into the casein micelles during the evaporation step in skim milk powder production. Subsequently, the reconstitution process of skim milk powder at 4, 20 and 40 °C was studied using turbidity. The turbidity decreased during the reconstitution period of 24 ​h, which was claimed to be due to the return of calcium from the colloidal phase to the serum. SDS-PAGE confirmed that the shift in salt equilibrium resulted in the migration of β-casein to the serum phase during reconstitution at 20 °C. However, no results were shown for the other temperatures studied. If the drop in turbidity is supposed to represent the migration of β -casein, it is unexpected that β-casein also migrates out of the casein micelles at 40 °C, taking the previously described theories on the temperature dependency of the equilibria of salts and β-casein into account (Oldfield et al., 2005; O’Connell et al., 2003). As the study by Martin et al. (2007) mostly utilizes indirect measurements that can be affected by polydispersity, it is not sure what the turbidity measurements exactly represent, and the dynamics of β-casein in reconstituted milk powders at different temperatures remains unclear.

Asymmetrical Flow Field-Flow Fractionation (AF4) (Wahlund et al., 2012; Wahlund and Giddings, 1987) in combination with various detectors has proven to provide detailed information about the properties of individual components in polydisperse food-related solutions and dispersions (Nilsson, 2013). This method has been applied earlier to macromolecular and colloidal components in fresh milk (Glantz et al., 2010; Guyomarc’h et al., 2010; Silva et al., 2013) and reconstituted skim milk (Lie-Piang et al., 2021). For reconstituted milk, the authors concluded that the casein micelle population contained larger components than the casein micelle population in fresh milk. They also found that the concentration of casein micelles increased during the first 20 ​min of reconstitution of skim milk powder at 50 °C. Hence, AF4 coupled to multiangle light scattering (MALS), UV, and refractive index (RI) detectors is a method that can deliver direct information on the properties of the casein micelles as well as reconstitution-related changes over time. The casein micelle concentration was determined from the RI response. A closer look into the data showed that the response of the UV and MALS detectors were not increasing proportionally with the RI response. Therefore, it was hypothesized that another phenomenon was occurring that could be related to the migration of β-casein between the serum and micellar phase and that AF4 coupled to multiple detectors could be a promising method to investigate the migration of β-casein between the micellar and the serum phase.

Therefore, the aim of the current study is to revisit the dynamics of β-casein dissociation and association to casein micelles in reconstituted skim milk using AF4. Initially, the dynamics were investigated using AF4 coupled to multiangle light scattering (MALS) and additional detectors such as UV, refractive index (RI), and fluorescence (FL). Subsequently, findings on the mass, conformation, and apparent density of the casein micelle population were used to investigate the distribution of mass in the casein micelles. To assess the influence of temperature on the dynamics, reconstitution of skim milk powder at cold (6 ​°C) and elevated temperatures (50 ​°C) were investigated.

## 2 Materials and methods

### 2.1 Materials

The skim milk powder (SMP) used in this study was agglomerated and medium heat treated and was supplied by Arla Foods Ingredients (Viby, Denmark). The powder was stored in an opaque and airtight container at room temperature. The humidity level was controlled and was always less than 35% relative humidity. The fresh ‘minimjölk’ (skim milk with <0.1% fat, pasteurized for 15 ​s at 72 ​°C) used in this study was from Skånemejerier (Malmö, Sweden). The fresh milk was always used at least four days before the expiry date and stored in a refrigerator at a temperature of 4 ​°C. The imidazole (Alfa Aesar, Haverhill, MA, USA), calcium chloride (VWR, Leuven Belgium), sodium azide (VWR), and hydrochloric acid (VWR) used for the AF4 carrier liquid were all analytical grade.

### 2.2 Sample treatment and hydration

The reconstituted skim milk samples were prepared by mixing 6 ​g of powder with 54 ​g of 0.02% sodium azide (VWR) in water (MQ-plus, Millipore, Burlington, MA, USA) to obtain a concentration of 10 ​wt%, chosen to correspond to the average total solids of skim milk. The water contained sodium azide as a preservative to prevent bacterial growth. Before mixing, the water was equilibrated to a temperature of 50 ​°C. The milk powder was dispersed in water using gentle agitation with a magnetic stirrer until the last powder particles had disappeared. Subsequently, the milk sample was mixed using an Ultra Turrax at 24 ​000 ​rpm (T25 Basic, IKA, Staufen im Breigau, Germany) for 1.5 ​min, to mimic the industrial process of a high shear mixer. The milk sample was reconstituted for a set time, with very slow magnetic stirring at approximately 120 ​rpm using a heating plate (RET basic, IKA, Staufen im Breigau, Germany), with a controlled temperature probe (ETS-D4 Fuzzy, IKA) to maintain the temperature at 50 ​°C. For the samples reconstituted at 6 ​°C, the dissolution procedure was the same, with the exception that the water used for reconstitution was cold on the addition of the milk powder. The sample was reconstituted and slowly stirred in a refrigerator at a temperature of 6 ​°C. Prior to analysis with AF4, samples were centrifuged for 2 ​min at 160 ​g to remove any cells, potential impurities, or undissolved particles. Very large particles (micron-sized and above) are not within the Brownian mode AF4 separation range (Myers and Giddings, 1982). To reduce the protein content and the viscosity, the milk samples was diluted 100x with of AF4 carrier liquid (described below) immediately before AF4 analysis.

For the casein dynamics experiments, β-casein from bovine milk (Sigma Aldrich, Steinheim, Germany) was labelled with fluorescein isothiocyanate (FITC) (Sigma Aldrich) based on the protocol of Twining (1984). 10 ​mg of β-casein was added to a 1 ​mL of 50 ​mM NaHCO_3_ (VWR) buffer containing 150 ​mM NaCl (VWR, Leuven Belgium) with a pH of 9.5. The mixture was gently stirred for 30 ​min. Subsequently, 1 ​mg of FITC was added and gently stirred for 30 ​min. To remove free FITC, the sample was purified using Vivaspin tubes with a membrane pore size of 10 ​kDa (Sigma Aldrich) at a speed of 13 ​400 ​rpm for 20 ​min. The last step was repeated until the filtered-out buffer was transparent.

All experiments and analyses were performed with unique replicates (n ​= ​2–5) and representative results are shown in the paper.

### 2.3 Asymmetrical flow field-flow fractionation

The asymmetrical flow field-flow fractionation (AF4) analysis was performed on an Eclipse 2 fractionation system (Wyatt technology, Dernbach, Germany) in connection with a 1100-series LC-system consisting of an ERC-3415 vacuum degasser (ERC, Riemerling, Germany), a G1311A pump, G1315A UV/VIS diode array detector and a G1329A auto sampler (Agilent technologies, Waldbronn, Germany). A Dawn Heleos II multi-angle light scattering (MALS) and Optilab t-Rex differential refractive index (RI) detector (Wyatt technology) were connected on-line after the channel. The UV-, MALS- and RI-detector operated at wavelengths of 250 ​nm, 664 ​nm, and 658 ​nm respectively. The fluorescence (FL) detector (FP-1520, Jasco, Tokyo, Japan) was also connected on-line and operated at excitation and emission wavelengths of 495 ​nm and 525 ​nm respectively.

Data collection was performed by Astra 6.1.2 (Wyatt technology). The asymmetrical flow field-flow fractionation channel was a Wyatt SC channel fitted with a 350 ​μm thick spacer. For all analyses, 10 ​kDa molecular weight cut-off membranes of regenerated cellulose (Merck-Millipore, Burlington, MA, USA) were used. The carrier consisted of 50 ​mM imidazole (Alfa Aesar), 2 ​mM calcium chloride (VWR) and 3 ​mM sodium azide (VWR) added to prevent microbial activity. The composition of the carrier liquid was based on a previous investigation where results were compared with those obtained by using real ultra-filtrate from milk as the carrier liquid (Glantz et al., 2010). The results showed excellent agreement and, hence, artefacts induced from the carrier liquid could be ruled out. The pH was adjusted to pH 6.7 with hydrochloric acid (VWR). Fractionation was run at ambient temperature (22 ​°C). Performance testing of the AF4-separation, as well as the MALS-RI detection and molar mass determination, was done by analysing solutions of bovine serum albumin (BSA, Sigma-Aldrich).

The AF4 method used a detector flow rate of 0.50 ​mL/min. Before injection was started, the system was allowed to stabilize crossflows and pressures for 1 ​min. The injection flow rate was 0.20 ​mL/min and the injection time was 2 ​min. The focusing/relaxation time was 3 ​min. The crossflow rate during injection and focusing was 1.0 ​mL/min. At the onset of the elution phase, the crossflow rate was 1.0 ​mL/min, which was kept constant for 4 ​min, thereafter, exponentially decaying with a t_½_ ​= ​2 ​min according to

(1) $$Q_{c}\left(t\right)=Q_{c,0}\cdot 2^{-\frac{t}{t_{1/2}}}$$

where Q_c, 0_ is the volumetric crossflow rate at the onset of the decay, t is the time, and t_½_ is the half-life of the volumetric crossflow. When the crossflow rate reached 0.15 ​mL/min the decay was stopped and a crossflow rate of 0.15 ​mL/min was kept for 50 ​min. Sample injection volume was 50 ​μL giving a sample mass load of 50 ​μg.

### 2.4 Data evaluation

AF4-MALS-UV-FL-RI data was evaluated using Astra 6.1.2 (Wyatt technology). The RI detector shows a non-linear background response (caused by the decaying crossflow), which is compensated by subtracting the RI response from a blank analysis. The molar mass calculations (when possible) were performed utilizing a first-order fit to the scattering detectors 5–11 (35–90°) according to the Berry method. Parameters for the sample were a refractive index increment, dn/dc, of 0.185 ​mL ​g^−1^ and a UV-extinction coefficient of 0.66 mL/mg^−1^·cm^−1^. It should be noted that the extinction coefficient is different for each protein since it depends on the UV absorbance of specific amino acids. Similarly, the dn/dc is also different for the individual proteins, although the differences in dn/dc are typically smaller than in the extinction coefficient and the utilized values offer a generally good approximation for proteins. The second virial coefficient term was assumed to be negligible at the low concentrations present in the MALS-detector.

The hydrodynamic radius (R_h_) was determined from AF4 elution times, using the Stokes-Einstein equation

(2) $$r_{h,i}=\frac{k_{B}T}{6\pi \eta D_{i}}$$

where $k_{B}$ is the Boltzmann constant, T. is the absolute temperature, η is the dynamic viscosity of the solvent and $D_{i}$ is the diffusion coefficient of sample component *i*. In turn, $D_{i}$ is obtained from

(3) $$\frac{dz_{i}}{dt}=f\left(Q_{c}\right)D_{i}$$

where $z_{i}$ is the position of sample component *i* along the channel, and t is the time. Equation (3) is a highly condensed version of the full differential equation, of which the derivation of and solution procedure is described elsewhere (Håkansson et al., 2012). The separation channel height was determined for every channel assembly using a solution of BSA (Magnusson et al., 2012) and the determined channel heights were in the range of 258–325 ​μm.

### 2.5 Transmission electron microscopy

The size and structure of casein micelles were characterized with transmission electron microscopy (TEM) according to the method of Martin et al., (2007) and Lie-Piang et al. (2021). To fixate the liquid milk sample, 1 ml of the sample at room temperature was mixed with 1 mL 5% agarose (Sigma-Aldrich) in water at 42 °C. Subsequently, the mixture was allowed to set at room temperature for 1 min, after which the solidified agarose was cut into cubes of 1 mm^3^. Fixation of the samples was performed for 2 h in 3% glutaraldehyde (Agar Scientific Ltd., Stansted, UK) in a 0.1 M sodium cacodylate (Agar Scientific Ltd) buffer at pH 6.6. Next, 1% osmium tetroxide (Agar Scientific Ltd.) was used to post-fixate the samples for 1 h. ​The post-fixated samples were embedded through acetone (VWR Chemicals) in an AGAR 100 (R1031) epoxy resin (Agar Scientific Ltd.) and cut in sections with 50–100 nm thickness through a Leica UC7 Ultramicrotome (Leica Microsystems, Wetzlar, Germany). To facilitate visualization, uranyl acetate (Merck-Millipore) and lead citrate (Merck-Millipore) were used as counterstains. Lastly, the samples were viewed in a JEOL 1400 Plus at 100 kV (JEOL Ltd., Tokyo, Japan) and captured by a Matataki CMOS camera (JEOL Ltd., Tokyo, Japan).

## 3 Results and discussion

### 3.1 Detector responses from casein micelles during reconstitution

An earlier study on the macromolecular and colloidal components of reconstituted milk showed that the response of the UV and MALS detectors for the casein micelle population were not increasing proportionally with the RI response after AF4 fractionation (Lie-Piang et al., 2021). Therefore, it was hypothesized that the phenomenon observed could be related to the migration of β-casein between the serum and micellar phase.

**Fig. 1:**
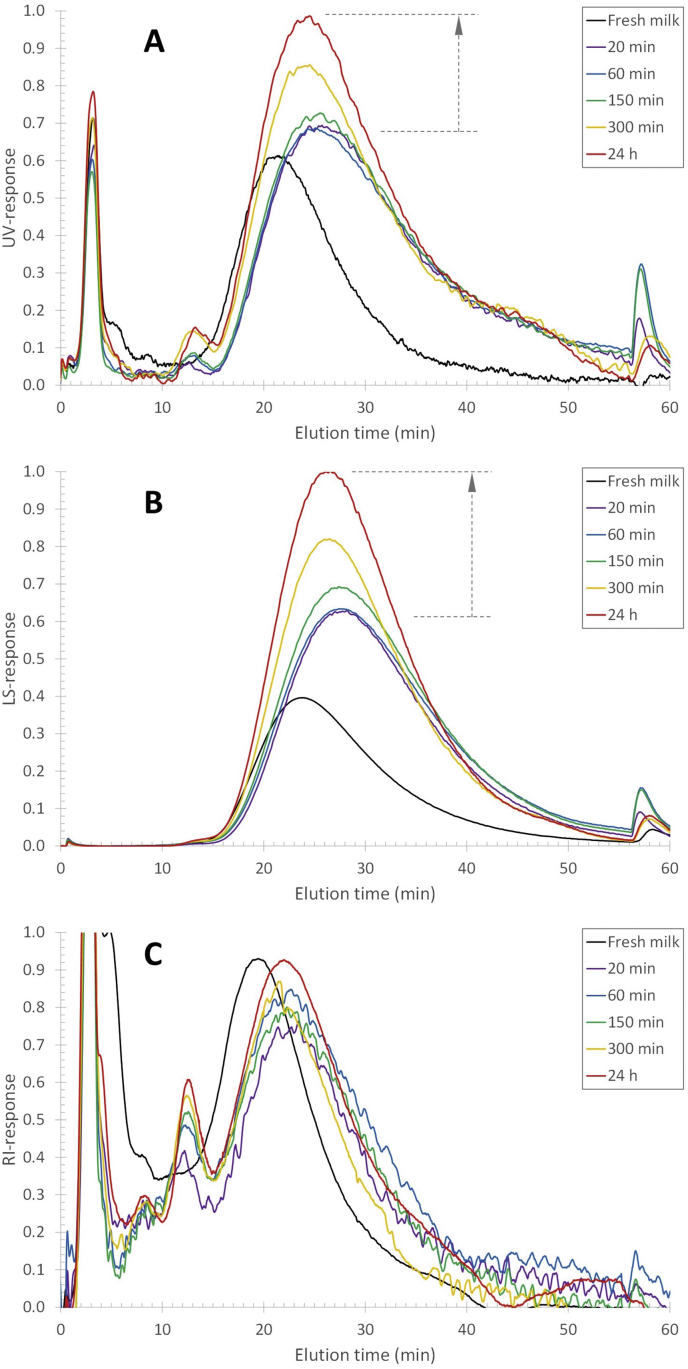
AF4-UV-MALS-RI fractograms of reconstituted skim milk after different hydration times at 50 ​°C. (A) UV response, (B) MALS 90° scattering response, and (C) RI response. The different coloured traces refer to the different reconstitution times as indicated in the legend.

Reconstituting skim milk powder at 50 ​°C leads to an increase in the response of the concentration-sensitive RI detector from ~0.27 (a.u.) at the casein population maximum to 0.31 (a.u.) after 24 ​h (Fig. 1C). The RI detector response is proportional to the concentration times the refractive index increment (*dn/dc*) (Equation (4)). The refractive index increment is a response factor that reflects the polarizability (van der Waals interactions) of the atoms and chemical groups constituting the analytes and, hence, the chemical composition in a given solvent.

(4) $$S_{RI}\propto \frac{dn}{dc}\cdot c$$

It is widely recognized that for most proteins, *dn/dc* does not differ significantly (Babul and Stellwagen, 1969; Le Févre, 1965; Zhao et al., 2011) and normally falls within a narrow range (0.18–0.19 ​mL/g). Thus, based on the RI response it can be concluded that the concentration of casein micelles increases with less than 15% over the reconstitution time range from 20 ​min to 24 ​h. In contrast, the UV and MALS responses increase more significantly within the reconstitution time of 20 ​min–24 ​h. The UV response of the same milk sample at the casein population maximum (at ~25 ​min elution time) increases from a response of 0.7–1.0 (Fig. 1A). Similarly, the MALS response increases from 0.63 to 1.0 (Fig. 1B). Clearly, there are additional phenomena occurring in the milk sample that affect the disproportional increase of 40%, or more, of the UV and MALS detector response, which do not increase the concentration of casein micelles proportionally (RI response increase <15%). This phenomenon also does not affect the hydrodynamic size of the casein micelles as the elution times stay essentially unchanged.

The response of the different detectors used (UV, MALS, and RI) are sensitive towards different properties of the sample. The UV-detector response is proportional to the concentration (*c*) times the molar absorptivity coefficient (*ε*) of the components at the wavelength used (in this study 250 ​nm) (Equation (5)).

(5) $$S_{UV,\;250}\propto \varepsilon_{250}\cdot c$$

The molar absorptivity coefficient is a response factor that is dependent on the chemical structure of the components. In general, proteins have a high molar absorptivity coefficient at 250 ​nm due to the aromatic amino acids (phenylalanine, tryptophan, tyrosine) common among the structural elements of proteins. For the MALS detector, the response is proportional to the weight-average molar mass (*M*_*w*_) of the components in an eluting fraction times the concentration of the components (Equation (6)).

(6) *S*_*MALS*_*∝ ​M*_*w*_*∙c*

Therefore, the significantly larger increase in UV and MALS responses must reflect some changes in composition and/or structure of the casein micelles. The increase in the MALS response indicates an increase in the mass of the casein micelles. The micelles become denser since the size (hydrodynamic size, as seen in the elution time) remains the same regardless of reconstitution time. Thus, some components of the reconstituted milk solution must adhere to the surface or enter the interior of the casein micelles as the reconstitution proceeds. That proteins, such as β-casein, can enter the interior of the casein micelles has been reported in the literature (Dalgleish and Corredig, 2012). The increase in UV response indicates that these components, associated with the casein micelles, must have a significant UV-activity at 250 ​nm. Also, the relatively large increase in UV and MALS responses implies that the components associated with casein micelles must be present in the reconstituted milk solution at a significant concentration. Considering the composition of reconstituted milk, it is likely that disproportional increase among the different detectors is due to the association of serum β-casein with the casein micelles.

### 3.2 Casein dynamics tracked by UV and MALS detector

**Fig. 2:**
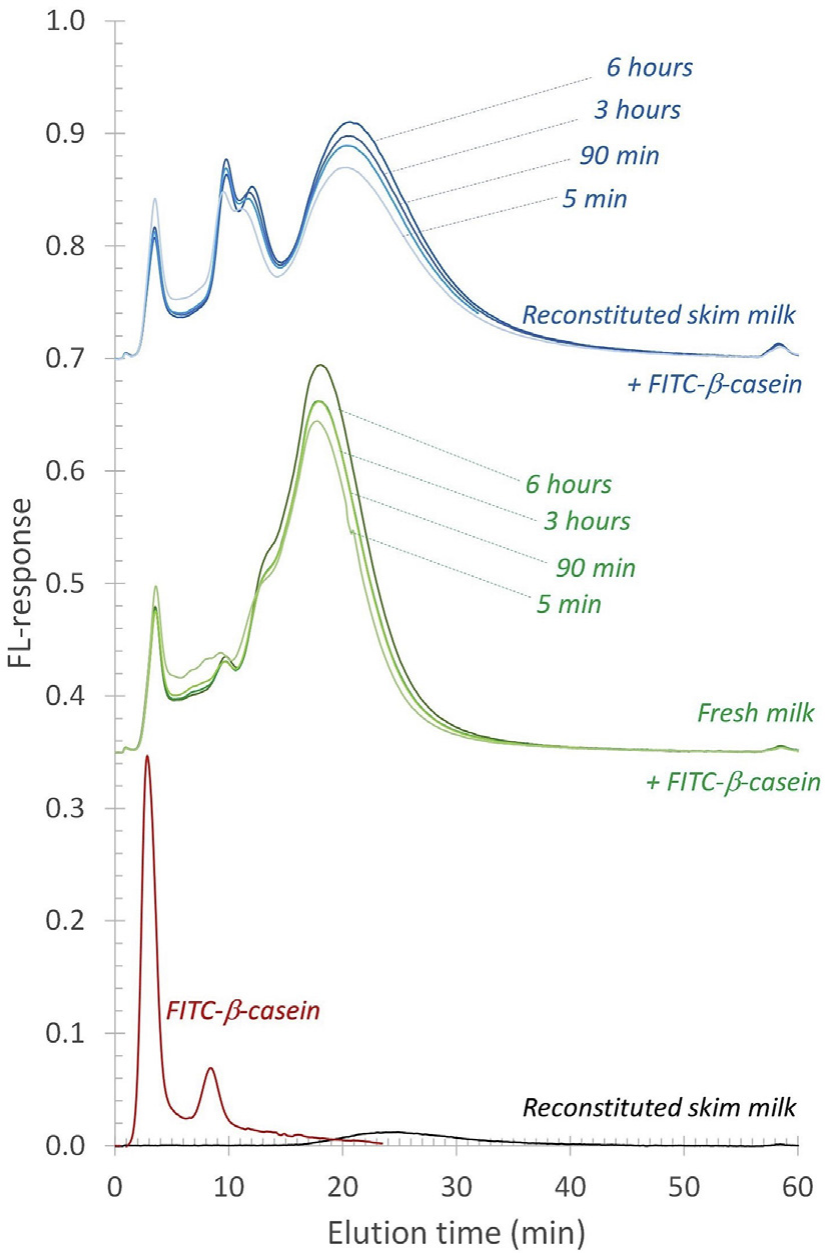
AF4-FL fractograms of reconstituted (blue traces) and fresh (green traces) milk after addition of FITC-labelled β-casein and FITC-labelled β-casein in the carrier liquid (red trace). The black trace represents the FL response of a reconstituted milk sample without anything added. The milk samples were incubated/reconstituted at 50 ​°C for 5, 95, and 365 ​min prior to analysis. (For interpretation of the references to colour in this figure legend, the reader is referred to the Web version of this article.)

Fluorescently labelled β-casein (FITC-β-casein) was added at the start of reconstitution of skim milk powder to confirm the migration of FITC-β-casein. The fluorescence (FL) detector coupled to the AF4 could selectively probe the FITC-β-casein in the complex milk sample. The same analysis was performed for heated fresh skim milk at 50 °C with the addition of FITC-β-casein to assess if the effect is dependent on the reconstitution process or not. The results of both milk samples (Fig. 2) show a broad elution profile with FL response from approximately 2-40 ​min. In both samples a peak is detected around 2–4 ​min, corresponding to an approximate hydrodynamic diameter of 3–6 ​nm, which is expected to be β-casein as it also is detected in the response of the pure FITC-β-casein (red trace). There is also a significant FL response of the single FITC-β-casein sample at elution times of 4–12 ​min (hydrodynamic diameter of 6–35 ​nm), an elution time range where one would expect larger components – such as oligomers or aggregated proteins - to elute. β-casein is known to self-assemble in solution and the components eluted in this elution time interval may be components that are at the onset of that phenomenon (Cragnell et al., 2017; de Kruif et al., 2012). This peak seems to be wider in the reconstituted milk sample, which could point to the association of FITC-β-casein to the aggregated protein population present at the corresponding elution time in this sample (Lie-Piang et al., 2021). Finally, in the elution time-range where the casein micelles are eluted (Lie-Piang et al., 2021), there is also a significant FL response for both the fresh and reconstituted milk samples in which FITC-β-casein was added. Given that the FL elution profile in the range 12–40 ​min match very well that of casein micelles, and no particles of this size eluted in the pure reference FITC-β-casein, it seems reasonable that the added FITC-β-casein associates with the casein micelles and that the MALS and UV detector are measuring this phenomenon.

**Fig. 3:**
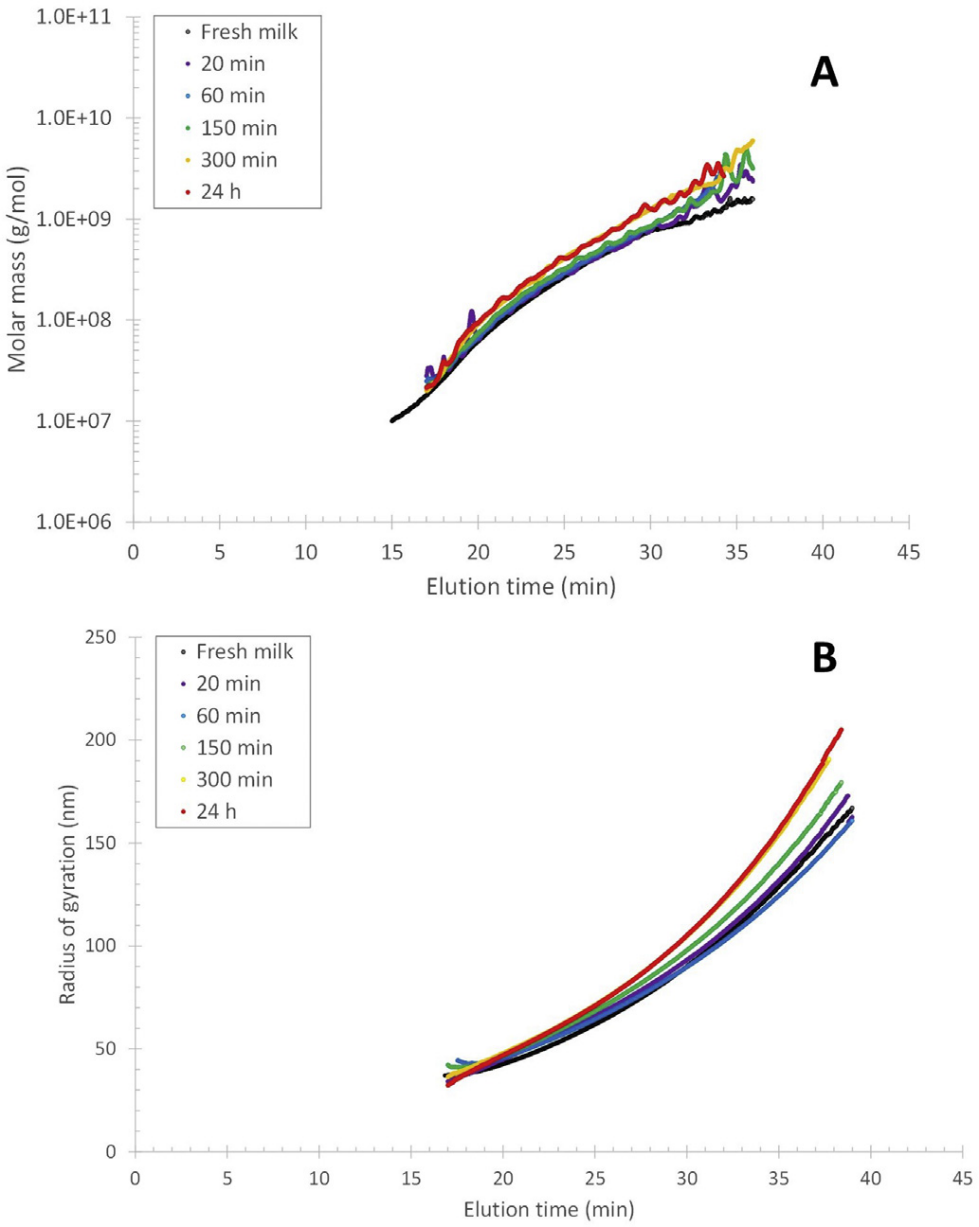
Molar mass (A) and radius of gyration (R_g_) (B) profiles for casein micelles after different reconstitution times of skim milk powder at 50 ​°C. Unprocessed fresh skim milk is added as a reference sample.

**Fig. 4:**
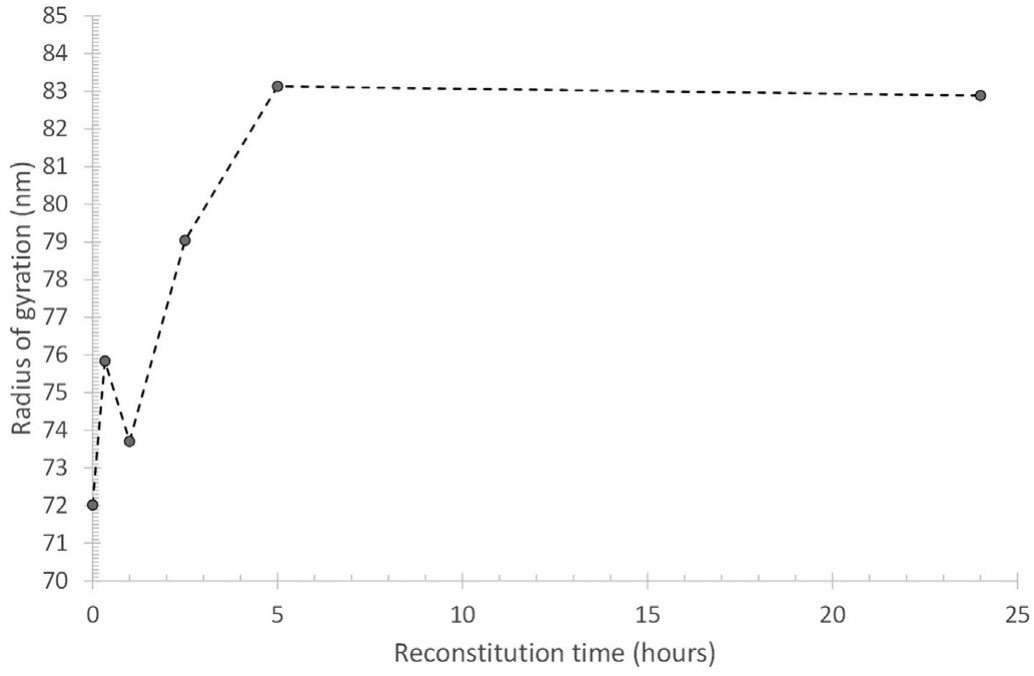
R_g_ for the casein micelles eluted at 27 ​min (R_h_ ​= ​96 ​nm) after different reconstitution times at 50 ​°C. The t_r_ ​= ​27 ​min correspond to the peak maximum in the MALS response (cf Fig. 1A). The time at zero minutes corresponds to the R_g_ of fresh milk.

The elution profile of the FITC-β-casein in fresh and reconstituted skim milk shows that already after 5 ​min incubation and reconstitution time, there is an FL response coinciding with the elution time of casein micelles (Fig. 2). It thus appears that β-casein migrates within 5 ​min to the casein micelles. For longer reconstitution/incubation times (6 ​h), the FL response in the 12–40 ​min elution range increases by approximately 17 and 24% for fresh and reconstituted milk respectively, compared to the response after 5 ​min (Fig. 2). The change in FL response is attributed to a small increase in the amount of FITC-β-casein associated with the casein micelles. There is also a corresponding decrease in smaller-sized FITC-β-casein as noted by a lower FL response (cf the samples of 5–90 ​min reconstitution time) in the 2–12 ​min elution time range. The data shows an initial association of FITC- β-casein to the casein micelles, occurring within 5 ​min. Secondly, there is a migration to the casein micelles that occurs within hours. The migration of mass to the micelles is also reflected in an increase in the radius of gyration (R_g_) (Fig. 3B) of the casein micelles in reconstituted skim milk, which is a measure of a change in mass located farther from the centre of mass of the object. The R_g_ at the peak maximum of casein micelles (elution time of 27 ​min, Fig. 1) as a function of reconstitution time, shows a vast increase until a plateau is reached after 5 ​h (Fig. 4). Thus, it seems that after 5 ​h of reconstitution, the migration of β-casein has reached an apparent equilibrium between the serum and micellar phase.

It should be noted that to enable these experiments the β-casein was chemically modified by the introduction of FITC to enable the selective monitoring of the β-casein in the complex matrix. As the FITC-group is relatively small (molar mass of 389 ​g/mol), in comparison to the larger β-casein (molar mass of approximately 24 ​000 ​g/mol), it can be argued that its impact on the overall protein properties should be relatively limited. However, the FITC-β-casein may still exhibit somewhat different chemical properties compared to non-labelled β-casein. Hence, the results should be treated as indicative of how un-labelled β-casein may behave.

### 3.3 Conformation and density of casein micelles

For now, it has been shown that the responses of the UV and MALS detectors are influenced by the migration of β-casein from the serum to the casein micelles, whereas the RI detector indicates the concentration change of the components analysed. By combining the responses from the different detectors, the molar mass, R_g_, apparent density, and conformation of the casein micelles can be calculated and related to the migration of β-casein into the casein micelles during the reconstitution of skim milk at 50 ​°C.

**Fig. 5:**
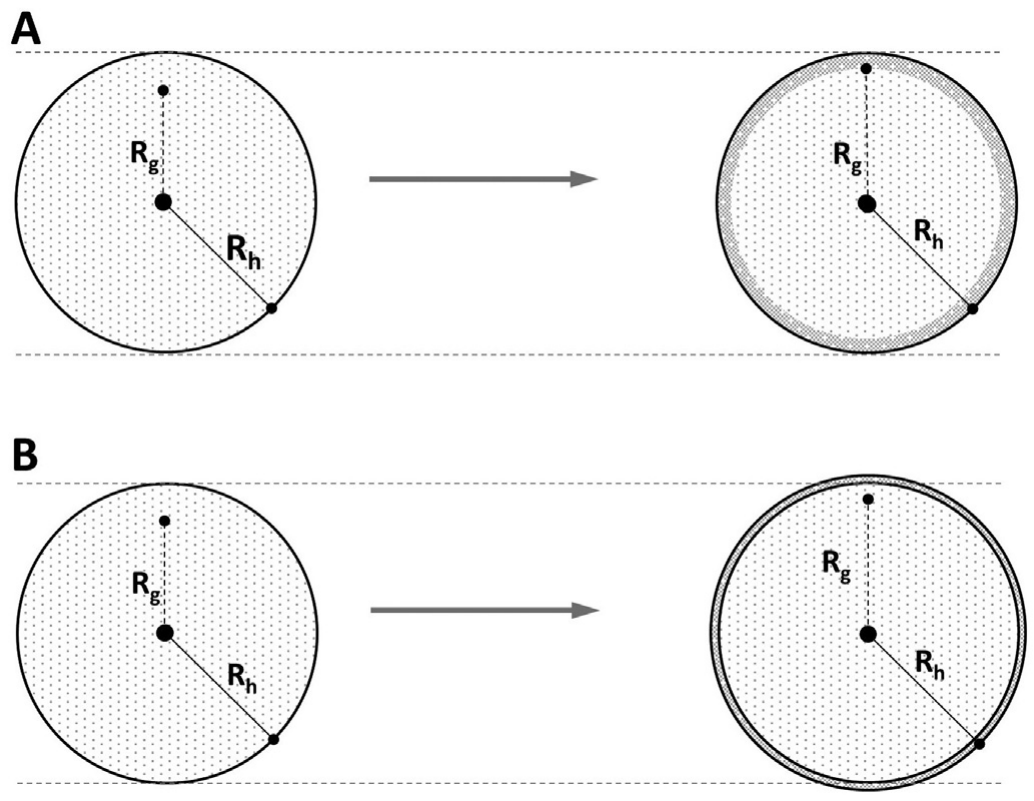
Two potential explanations for the migration of mass into the casein micelles as measured by the R_g_: A) the outer shell of the casein micelles becomes denser due to the migration of β-casein into the casein micelles while the R_h_ remains unchanged and B) β-casein adheres into a dense layer at the casein micelle surface, slightly increasing the R_h_. In both cases it is expected that the R_g_ would increase.

**Fig. 6:**
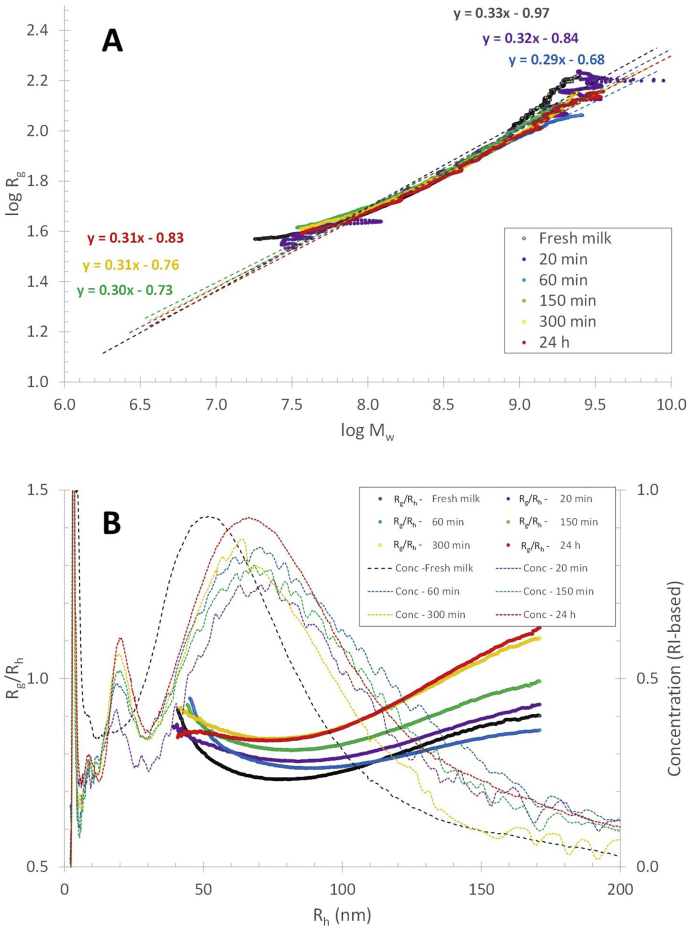
(A) Conformation plot and (B) radius ratio (R_g_/R_h_) and RI response (concentration) vs. hydrodynamic radius (R_h_) for casein micelles in skim milk powder after different reconstitution times at 50 ​°C.

The molar mass and R_g_ across the casein micelle population are shown in Fig. 3A and B. As can be expected, due to the migration of β-casein to the casein micelles, the molar mass of the casein micelle fraction increases (it should be noted that the y-axis is logarithmic) with reconstitution time. The R_g_ also increases, which indicates that there is an increase in mass located farther away from the centre of gravity of the casein micelle. Hence, either in the outer shell of the casein micelles (Fig. 5A) or rather adsorbed at the casein micelle surface (Fig. 5B). The ratio of R_g_ and the hydrodynamic radius (R_g_/R_h_) indicates the shape of objects such as particles or macromolecules (Nilsson, 2013), and also how the mass is distributed in the object. R_h_ was determined from the elution time and elution profile of the casein micelles and remain independent of reconstitution time (Fig. 1), which was also reported earlier (Martin et al., 2007; Lie-Piang et al., 2021). As the R_g_/R_h_ increases to approximately 0.9–1.4 after 24 ​h of reconstitution (Fig. 6B), the increase must be caused by an increase in R_g_. Hence, the components responsible for the increase in mass (the β-caseins) are located in the outer parts of the casein micelles as schematically illustrated in Fig. 5A.

**Fig. 7:**
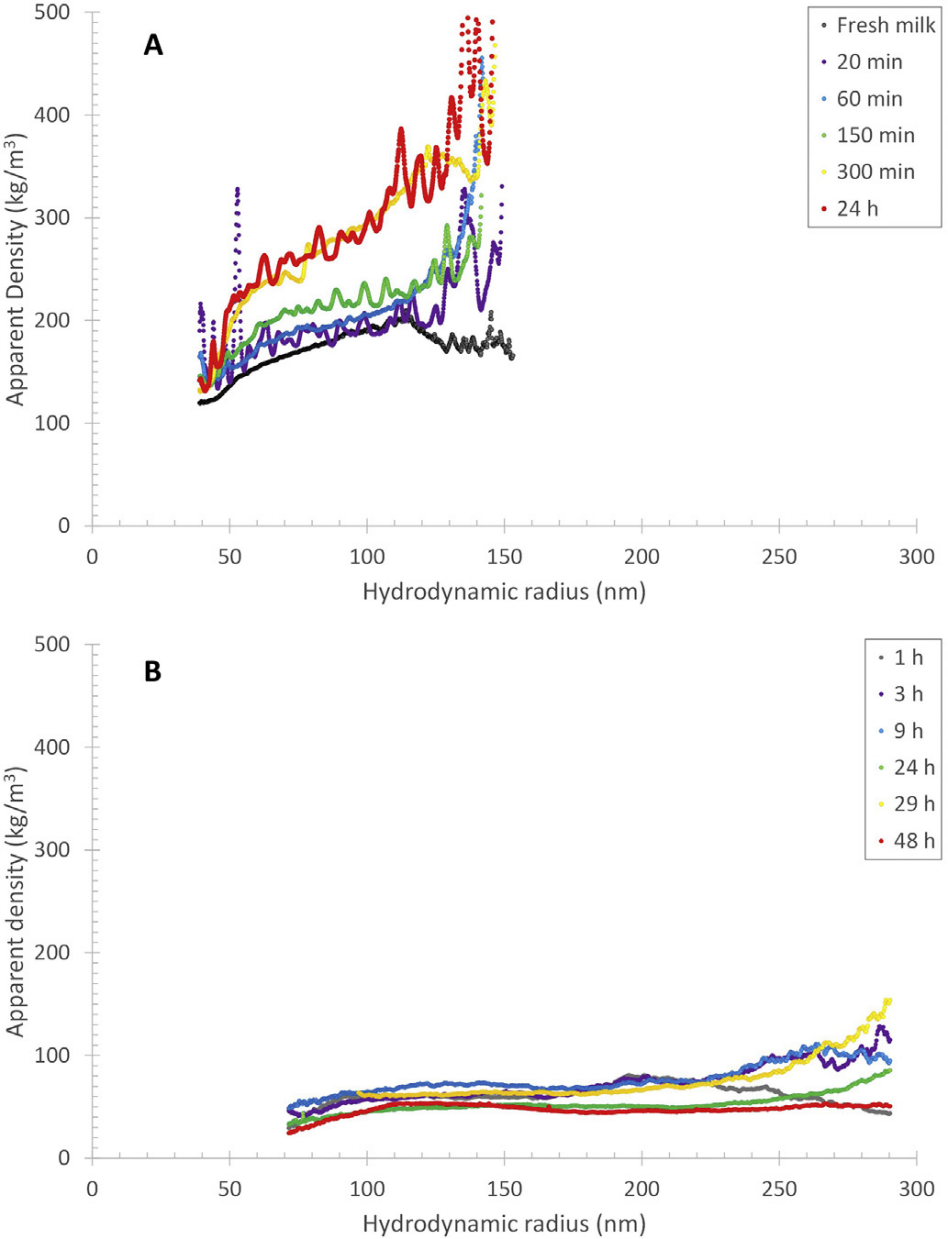
Apparent density vs. hydrodynamic radius (R_h_) for casein micelles after different reconstitution times (A) at 50 ​°C and (B) at 6 ​°C.

The combination of the molar mass and R_g_ data can deliver information on how the molar mass increases with the size of the components (i.e. the scaling relationship) which can be presented in a so-called conformation plot. The scaling relationship between molar mass and R_g_ for the casein micelle fraction (Fig. 6A) is close to the theoretical value for spheres, i.e. 0.33, independently of reconstitution time and, hence, migration of β-casein. As the casein micelles have spherical or close to a spherical shape, the *R*_*h*_ and the molar mass, *M*, can be used to calculate the apparent density, *ρ*_*app*_ (Glantz et al., 2010), over the size distribution as:

(7) $$\rho_{app}=\;\frac{m}{V}=\frac{\left(M/N_{A}\right)}{\left(\frac{4}{3}\pi \cdot R_{h}^{3}\right)}$$

where *m* is the mass, *V* is the volume and *N*_*A*_ is the Avogadro number (Fig. 7). As the casein micelles show an increase in mass but no change in hydrodynamic size, an increase in density is expected throughout the reconstitution. The apparent density for the casein micelles that elutes at 25 ​min (i.e. *R*_*h*_ ​= ​85 ​nm) show an increase from 180 ​kg/m^3^ at t ​= ​0, to 260 ​kg/m^3^ after 24 ​h of reconstitution. This can be attributed to an increase in mass caused by β-casein migration to the micellar phase. It should be noted that the apparent density is an underestimation of the real density of casein micelles, which has been reported as 1079 ​kg/m^3^ (de Kruif et al., 2012) and 1063 ​kg/m^3^ (Kirchmeier, 1973). This can be explained by the fact that MALS measures only the mass of the macromolecular components of the casein micelles and not the solvent in the structure. However, the volume assumes the casein micelles are spheres with solvent. This results in an overestimation of the volume. A correction for the water fraction in the casein micelles of approximately 79% (McMahon and Brown, 1984) results in a real density in the range of 850–1200 ​kg/m^3^, which are in the same range as values from the aforementioned literature. In summary, the combination of the responses of the detectors can provide further detailed information on the location of the mass within the casein micelles, as well as its density and conformation. There is a clear increase in density of the casein micelles upon reconstitution that can be attributed to the migration of β-casein from the serum into the outer shell of the casein micelles.

### 3.4 Comparison of casein micelles reconstituted at 6 ​°C and 50 ​°C

While the general recommendation is to reconstitute milk at elevated temperatures, milk powder is sometimes reconstituted at lower temperatures. With the proposed method from this study as well as the previous research (Lie-Piang et al., 2021), it is now possible to investigate the impact of a lower reconstitution temperature on the concentration, size, and dynamics of the β-casein in casein micelles.

**Fig. 8:**
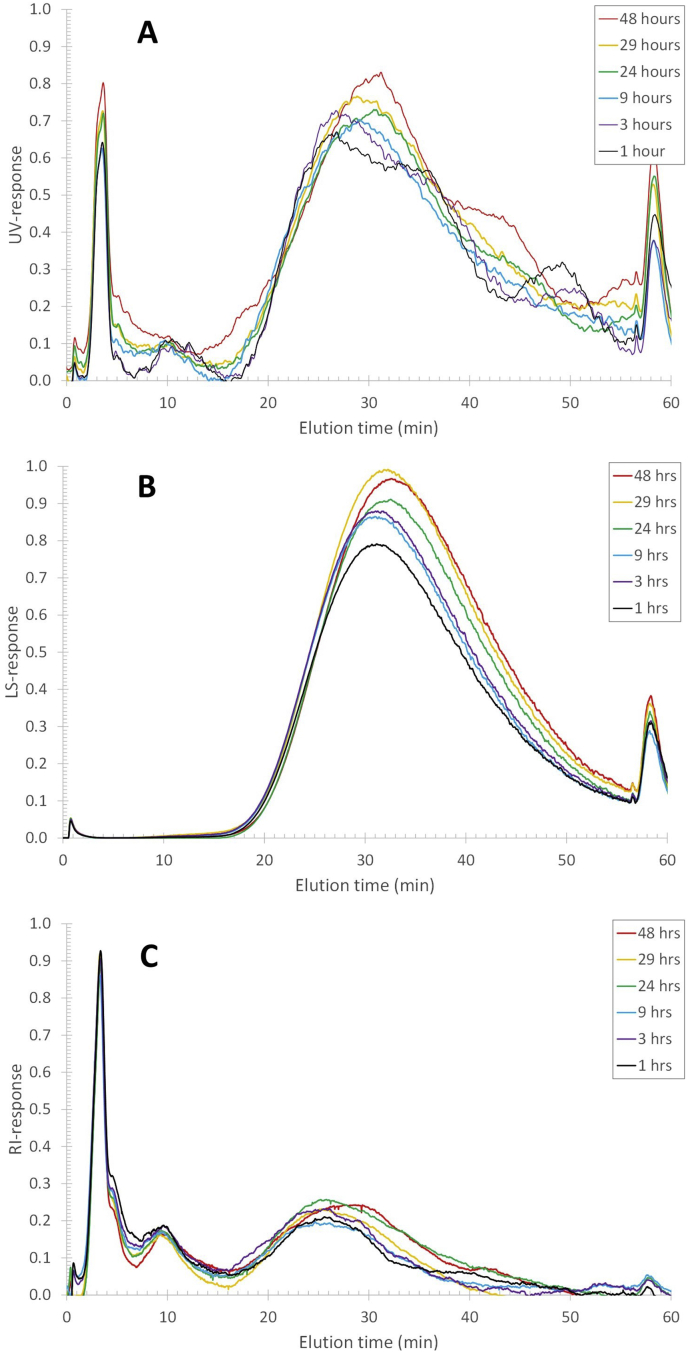
AF4-UV-MALS-RI fractogram of reconstituted skim milk after different reconstitution times at 6 ​°C. (A) UV response, (B) MALS 90° response, and (B) RI response. The different coloured traces refer to the different reconstitution times as indicated in the legend.

**Fig. 9:**
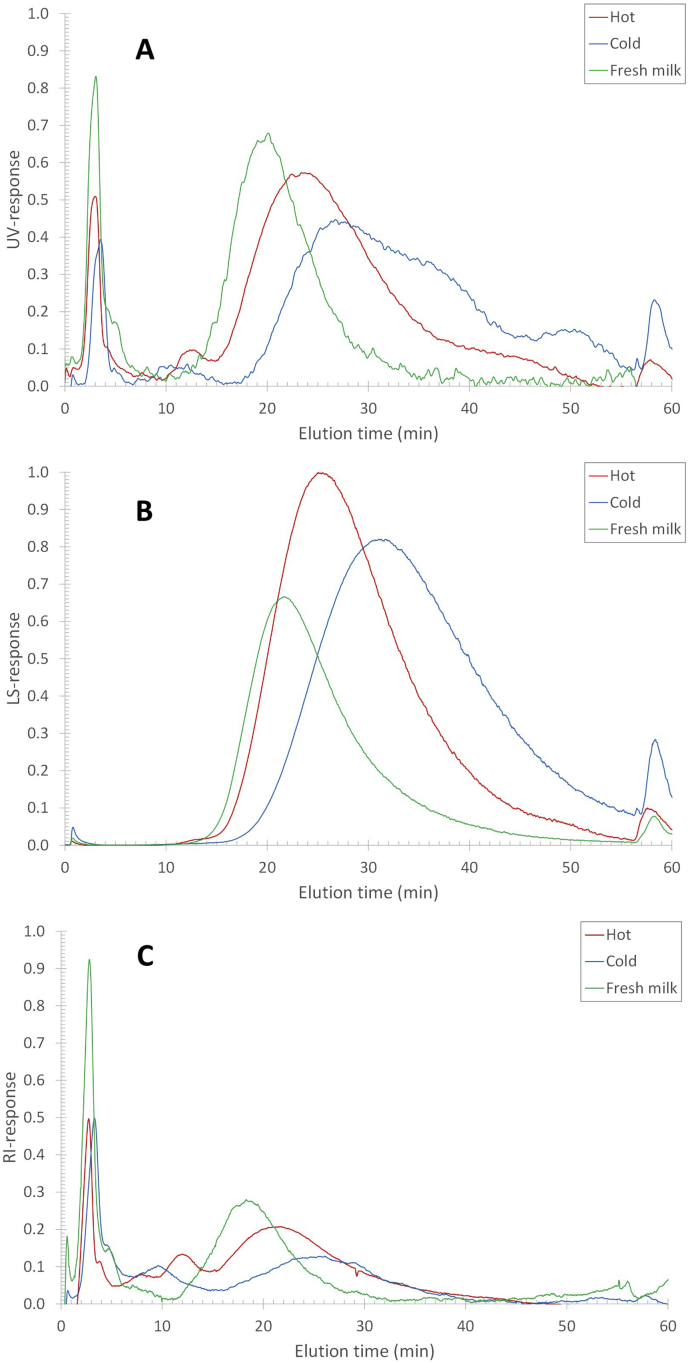
AF4-UV-MALS-RI fractogram of reconstituted skim milk after reconstitution at 6 ​°C (blue traces) and 50 ​°C (red traces) for 3 ​h. For reference the elution profile of fresh milk (green traces) is included. (A) UV response, (B) MALS 90° response, and (C) RI response. (For interpretation of the references to colour in this figure legend, the reader is referred to the Web version of this article.)

A dispersion of skim milk powder was reconstituted at 6 ​°C for 48 ​h (Fig. 8) and compared to skim milk reconstituted at 50 ​°C, and pasteurized fresh milk (Fig. 9). The results show that there is an increase in all detector responses with the reconstitution time at 6 ​°C. The increase in UV, LS, and RI response at peak maximum when reconstituting at 6 ​°C is more limited compared to reconstitution at 50 ​°C during reconstitution of 1–48 ​h. This is already observed after 3 ​h when comparing the warm and cold reconstitution and fresh milk (Fig. 9). Also, in contrast to the reconstitution at 50 ​°C, there is no differential increase in the UV and MALS responses compared to the RI as they all increase by roughly 20%. Therefore, no migration of β-casein can be detected. It is hypothesized that this because the β-casein proteins remain in the serum as a result of the increased solubility of calcium-phosphate clusters and a decrease in the hydrophobic effect at low temperatures (Creamer et al., 1977; Downey and Murphy, 1970; Pierre and Brule, 1981). If β-casein would migrate out of the casein micelles at this temperature, the UV and MALS response would increase with a lower rate compared to the RI response, which is not the case. The increase that is taking place in all detectors, thus, be attributed to a slow concentration increase of individual casein micelles, as was observed previously (Lie-Piang et al., 2021).

**Fig. 10:**
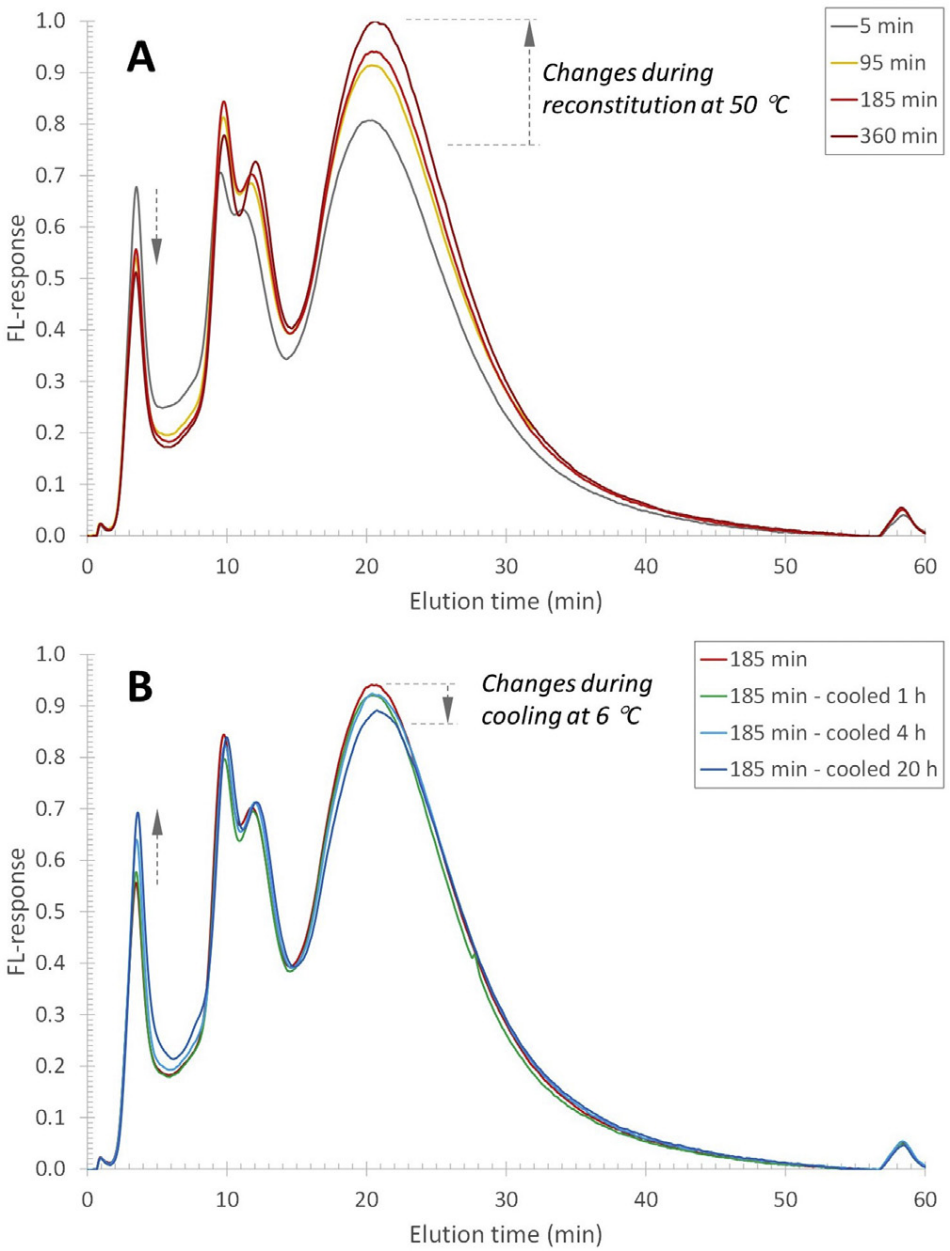
AF4-FL fractogram of reconstituted skim milk after addition of FITC-labelled β-casein that was initially reconstituted at 50 ​°C for 5, 95 and 185 ​min (A, red traces), and subsequently cooled to 6 ​°C for 20 ​h (B, blue traces). (For interpretation of the references to colour in this figure legend, the reader is referred to the Web version of this article.)

As the migration of β-casein from micelles to serum is temperature-dependent and reversible (Dalgleish, 2011), another skim milk powder sample was initially reconstituted at 50 ​°C for 185 ​min with the addition of FITC-β-casein, after which it was cooled down to 6 ​°C for 20 ​h (Fig. 10). The purpose was to confirm that the method can track the reversibility of the temperature-dependent migration of β-casein. The FL response for the casein micelle population, eluting around 25 ​min, increases upon reconstituting at 50 ​°C for 185 ​min. When the milk sample is subsequently cooled to 6 ​°C for 20 ​h, there is a small decreasing trend in the FL response in the casein micelle population. Hence, β-casein should be migrating out from the micellar phase. This trend is verified in the first peak in Fig. 10, identified as non-micellar β-casein, which is increasing upon reconstitution at 6 ​°C. These results show that the process is reversible and that the method can track FITC-β-casein migrating from the casein micelles to the serum at 6 ​°C.

**Fig. 11:**
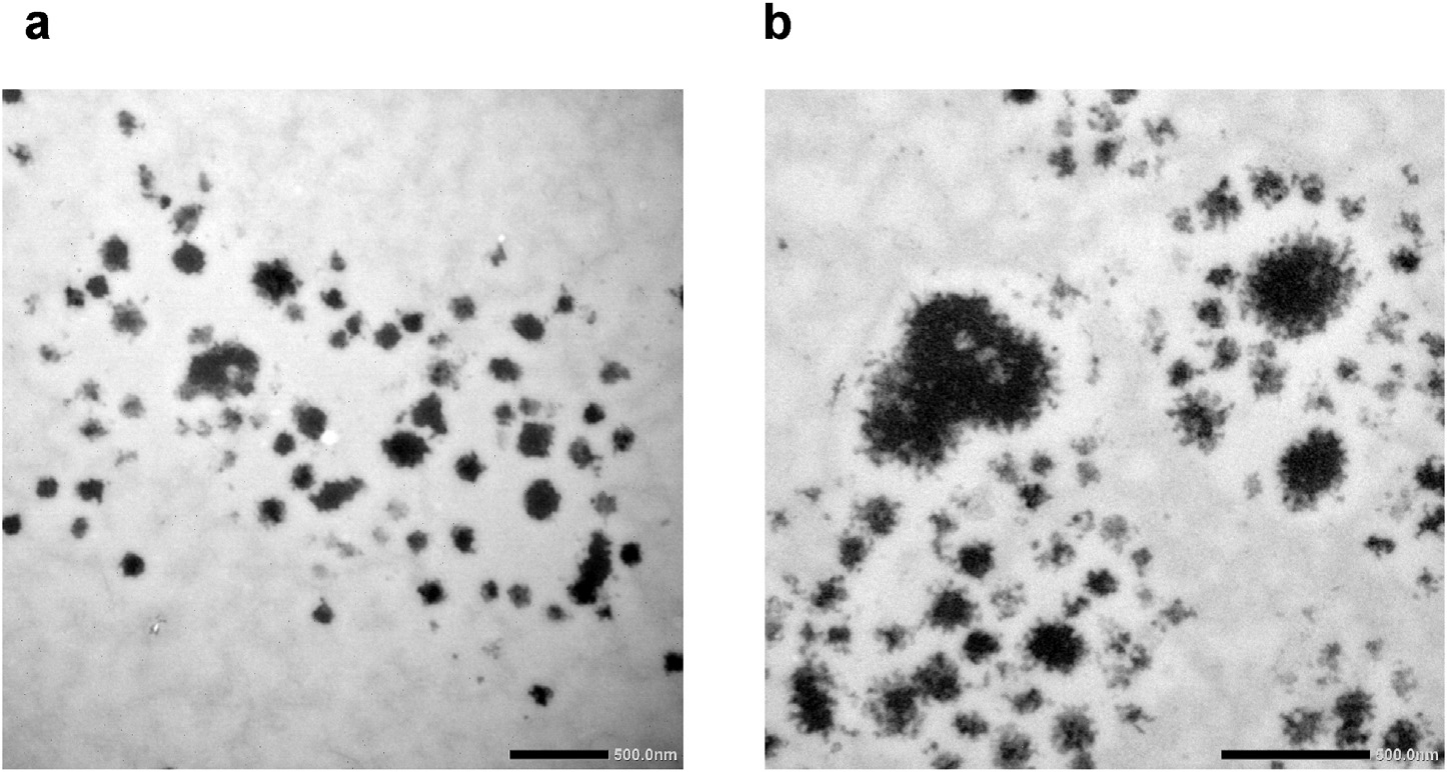
TEM images of casein micelles in reconstituted skim milk at (A) 50 ​°C for 1 ​h (x10k magnification) and (B) 6 ​°C for 24 ​h (x15k magnification). The scale bar is 500 ​nm.

The constant elution time shows that the hydrodynamic size of the casein micelles in skim milk powder reconstituted at 6 ​°C is not significantly impacted by reconstitution time in the investigated range (1–48 ​h) (Fig. 8), as was found for the reconstitution at 50 ​°C (Fig. 1). However, the casein peak maximum (as detected on the UV) is at 27 ​min (R_h_ ​= ​90 ​nm) for the sample reconstituted at 6 ​°C, while for the skim milk powder reconstituted at 50 ​°C the peak maximum is at 24 ​min (R_h_ ​= ​72 ​nm), and for the fresh milk the peak maximum is at 20 ​min (R_h_ ​= ​51 ​nm) (Fig. 9). The entire casein micelle size distribution is, thus, shifted towards longer elution times (i.e. larger sizes) than for fresh milk and when the skim milk powder is reconstituted at 6 vs. 50 ​°C. Reconstituting for longer times (up to 48 ​h) does not change this. The question of why the R_h_ of the casein micelle fraction is larger when reconstituting at 6 ​°C cannot be explicitly explained based on the obtained data. It is speculated that the casein micelles are more loosely associated at low temperatures due to the liberation of calcium-phosphate clusters from the casein micelles to the serum. This is attributed to the higher solubility of the calcium-phosphate clusters at lower temperatures (Pierre and Brule, 1981; Law and Leaver, 1998). That the casein micelles are more loosely associated is also reflected in the apparent density of casein micelles in cold reconstituted skim milk, which is substantially lower compared to the casein micelles reconstituted at 50 ​°C (Fig. 7B). A comparison of TEM images of casein micelles reconstituted at 50 ​°C (after 1 ​h of reconstitution) and 6 ​°C (24 ​h of reconstitution) also clearly showed that the casein micelles are more loosely associated and fuzzy at low temperatures, especially at the micelle surface (Fig. 11).

## 4 Conclusion

AF4-MALS-UV-FL-RI was used to revisit the dynamics of β-casein during the reconstitution of skim milk powder at 6 and 50 ​°C. The RI was found to measure the concentration increase of casein micelles during reconstitution, whereas the MALS and the UV response represent a change of mass occurring in the casein micelle. Using FITC-β-casein in combination with fluorescence detection, showed that the MALS and UV response track β-casein migrating in and out of the casein micelles in both fresh and reconstituted milk. At 50 ​°C, the β-casein was proven to migrate to the casein micelles for the initial 5 ​h of reconstitution. As a result, the apparent density of the casein micelles increased as well. The mass that migrated to the casein micelles was found to be located in the outer shell of the casein micelles rather than in the centre or at the surface. The migration of β-casein could be reversed upon cooling the same sample to 6 ​°C. The concentration of casein micelles increased at a lower rate than when reconstituting skim milk powder at 6 ​°C compared to 50 ​°C. However, the MALS and UV responses showed a similar increase compared to the RI response and, hence, no or very limited migration of β-casein takes place. This can be attributed to a shift in equilibrium of β-casein to the serum phase due to increased solubility of calcium-phosphate clusters and a decreased hydrophobic effect at 6 ​°C. Moreover, reconstitution of skim milk powder at 6 ​°C yields casein micelles of a larger size than when reconstituting at 50 ​°C. It is hypothesized that the higher solubility of calcium phosphate at lower temperatures could be responsible for this difference in size. β-casein migration was observed while incubating fresh skim milk as well as for reconstituted skim milk and, thus, it could be concluded that migration of β-casein is not only a phenomenon of fresh milk but rather occurs in milk in general. This study has proven the potential of using AF4 and a multi-detector approach to unravel detailed information about casein micelles in both reconstituted and fresh milk. More specifically, information on the concentration, size, density, and mass distribution within the casein micelles was presented to increase the understanding of the dynamics of casein micelles and β-casein.

## CRediT authorship contribution statement

**Anouk Lie-Piang:** Conceptualization, Formal analysis, Investigation, Methodology, Validation, Writing – original draft, Writing – review & editing. **Mats Leeman:** Conceptualization, Formal analysis, Methodology, Supervision, Writing – original draft, Writing – review & editing. **Alejandra Castro:** Formal analysis, Methodology, Writing – original draft. **Erik Börjesson:** Conceptualization, Methodology, Project administration, Supervision, Writing – review & editing. **Lars Nilsson:** Conceptualization, Methodology, Project administration, Supervision, Writing – review & editing.

## Declaration of competing interest

The authors declare that they have no known competing financial interests or personal relationships that could have appeared to influence the work reported in this paper.
